# Genome Sequence of the Probiotic Strain *Lactobacillus rhamnosus* (Formerly *Lactobacillus casei*) LOCK900

**DOI:** 10.1128/genomeA.00640-13

**Published:** 2013-08-15

**Authors:** Tamara Aleksandrzak-Piekarczyk, Anna Koryszewska-Bagińska, Jacek Bardowski

**Affiliations:** Institute of Biochemistry and Biophysics, Polish Academy of Sciences, Warsaw, Poland

## Abstract

*Lactobacillus rhamnosus* LOCK900 fulfills the criteria required for probiotic strains. In this study, we report a whole-genome sequence of this isolate and compare it with other *L. rhamnosus* complete genome sequences already published.

## GENOME ANNOUNCEMENT

*Lactobacillus rhamnosus* strain LOCK900 (formerly *Lactobacillus casei* LOCK900; patent no. 209988) was originally isolated from feces of a healthy 26-year-old woman and was obtained from the Pure Culture Collection of the Technical University, Lódz, Poland. The new species affinity of this strain was identified in this study based on the sequences of genomic markers, such as 16S rRNA, *rpoA*, and *pheS* genes (1).

It has been shown that *L. rhamnosus* LOCK900 fulfills the criteria required for probiotic strains, such as resistance to high concentrations of bile salts and low pH (2, 3). Also, its ability to adhere to cells of the Caco-2 epithelial cell line has been reported (4). In addition, its antiallergic potential has been manifested by induction of T_h_1 cytokines (interleukin 12 [IL-12], IL-18, gamma interferon [IFN-γ], and tumor necrosis factor alpha [TNF-α]) and regulatory transforming growth factor β_1_ (TGF-β_1_) in blood cell cultures of allergic children (5). Lastly, no pro-allergic cytokines were found after bacterial stimulation (5).

The whole genome of *L. rhamnosus* LOCK900 was sequenced using the GS FLX Titanium platform (Roche). Paired and unpaired reads obtained with 81-fold genomic coverage were assembled using Newbler *de novo* assembler (Roche) software. The final circularized genome molecule of 2,883,376 bp with a G+C content of 46.8% was deciphered.

Genome annotation was performed by merging the result from the RAST (Rapid Annotation using Subsystem Technology) server (6) and tRNAscan-SE (7) and followed by manual inspection, which predicted 2,838 coding sequences (CDS), 59 tRNAs, and 5 rRNA loci. Of the total number of CDS found, approximately 760 are hypothetical proteins with unknown functions. No plasmids were found in this isolate, which is relatively common for lactobacilli and different from lactococci (8).

The analysis retrieved from the RAST server revealed 310 subsystems existing in *L. rhamnosus* LOCK900 and revealed the absence of subsystem features for photosynthesis, iron acquisition and metabolism, motility, and chemotaxis, which has also been observed in all other completely sequenced genomes of this species (9–11). Due to the genome size reduction compared with other fully sequenced strains of *L. rhamnosus*, LOCK900 displaces a reduced number of genes distributed in individual categories, particularly carbohydrates, cell wall and capsule, virulence, disease, and defense. *L. rhamnosus* LOCK900 also showed a lack of many genes distributed in the categories of phages and prophages; however, the analysis performed via the PHAST (Phage Search Tool) server (12) indicated the existence of one intact and one incomplete prophage within its genome. On the other hand, despite the LOCK900 reduced genome, many genes are more abundantly represented, mostly in the categories of protein metabolism, RNA metabolism and regulation, and cell signaling.

Comparative genomic analysis of *L. rhamnosus* LOCK900 with four published *L. rhamnosus* complete genome sequences showed it has the greatest similarity to *L. rhamnosus* Lc705 and *L. rhamnosus* ATCC 8530 and less similarity to the probiotic isolate *L. rhamnsosus* GG. In comparison to *L. rhamnosus* Lc705 and ATCC 8530, this analysis showed the presence of CRISPR-associated proteins and the existence of a complete type II restriction-modification (RM) system consisting of a restriction endonuclease and modification methylase EcoRV in the *L. rhamnosus* LOCK900 genome.

### Nucleotide sequence accession number.

The complete sequence of the *L. rhamnosus* LOCK900 genome is deposited in GenBank under the accession number CP005484.

## References

[1] Naser SM Dawyndt P Hoste B Gevers D Vandemeulebroecke K Cleenwerck I Vancanneyt M Swings J 2007 Identification of lactobacilli by *pheS* and *rpoA* gene sequence analyses. Int. J. Syst. Evol. Microbiol. 57:2777–2789 18048724 10.1099/ijs.0.64711-0

[2] Cukrowska B Motyl I Kozáková H Schwarzer M Górecki RK Klewicka E Śliżewska K Libudzisz Z 2009 Probiotic *Lactobacillus* strains: in vitro and in vivo studies. Folia Microbiol. (Praha) 54:533–537 20140722 10.1007/s12223-009-0077-7

[3] Motyl I 2002 Ph.D. thesis Technical University of Lodz, Lódz, Poland

[4] Marewicz E Moneta J Motyl I Heczko P Libudzisz Z 2000 Comparison of adherence to Caco-2 human epithelial cell line of *Lactobacillus* and *Bifidobacterium* strains of various origin: human, animal and vegetal. Med. Sci. Prompt. 54(Suppl. 3):34

[5] Cukrowska B Rosiak I Klewicka E Motyl I Schwarzer M Libudzisz Z Kozakova H 2010 Impact of heat-inactivated *Lactobacillus casei* and *Lactobacillus paracasei* strains on cytokine responses in whole blood cell cultures of children with atopic dermatitis. Folia Microbiol. (Praha) 55:277–280 20526842 10.1007/s12223-010-0041-6

[6] Aziz R Bartels D Best A DeJongh M Disz T Edwards R Formsma K Gerdes S Glass E Kubal M Meyer F Olsen G Olson R Osterman A Overbeek R McNeil L Paarmann D Paczian T Parrello B Pusch G Reich C Stevens R Vassieva O Vonstein V Wilke A Zagnitko O 2008 The RAST server: Rapid annotations using subsystems technology. BMC Genomics 9:75. 10.1186/1471-2164-9-75 18261238 PMC2265698

[7] Schattner P Brooks AN Lowe TM 2005 The tRNAscan-SE, snoscan and snoGPS web servers for the detection of tRNAs and snoRNAs. Nucleic Acids Res. 33:W686–W689. 10.1093/nar/gki366 15980563 PMC1160127

[8] Górecki RK Koryszewska-Bagińska A Gołębiewski M Żylińska J Grynberg M Bardowski JK 2011 Adaptative potential of the *Lactococcus lactis* IL594 strain encoded in its 7 plasmids. PLoS One 6:e22238. 10.1371/journal.pone.0022238 21789242 PMC3138775

[9] Kankainen M Paulin L Tynkkynen S von Ossowski I Reunanen J Partanen P Satokari R Vesterlund S Hendrickx APA Lebeer S De Keersmaecker SCJ Vanderleyden J Hämäläinen T Laukkanen S Salovuori N Ritari J Alatalo E Korpela R Mattila-Sandholm T Lassig A Hatakka K Kinnunen KT Karjalainen H Saxelin M Laakso K Surakka A Palva A Salusjärvi T Auvinen P de Vos WM 2009 Comparative genomic analysis of *Lactobacillus rhamnosus* GG reveals pili containing a human-mucus binding protein. Proc. Natl. Acad. Sci. U. S. A. 106:17193–17198 19805152 10.1073/pnas.0908876106 PMC2746127

[10] Morita H Toh H Oshima K Murakami M Taylor TD Igimi S Hattori M 2009 Complete genome sequence of the probiotic *Lactobacillus rhamnosus* ATCC 53103. J. Bacteriol. 191:7630–7631 19820099 10.1128/JB.01287-09 PMC2786603

[11] Pittet V Ewen E Bushell BR Ziola B 2012 Genome sequence of *Lactobacillus rhamnosus* ATCC 8530. J. Bacteriol. 194:726–726 22247527 10.1128/JB.06430-11 PMC3264073

[12] Zhou Y Liang Y Lynch KH Dennis JJ Wishart DS 2011 PHAST: a fast phage search tool. Nucleic Acids Res. 39:W347–W352. 10.1093/nar/gkr485 21672955 PMC3125810

